# Wetting of MXenes and Beyond

**DOI:** 10.1007/s40820-023-01049-x

**Published:** 2023-04-30

**Authors:** Massoud Malaki, Rajender S. Varma

**Affiliations:** 1 Department of Mechanical Engineering, Isfahan University of Technology, Daneshgah e Sanati Hwy, Khomeyni Shahr, Isfahan, 84156-83111 Iran; 2 Regional Centre of Advanced Technologies and Materials, Palacký University in Olomouc, Šlechtitelů 27, 783 71 Olomouc, Czech Republic

**Keywords:** MXene, Wetting, Hydrophilicity, Composites, 2D material

## Abstract

MXenes are a class of 2D nanomaterials with exceptional tailor-made properties such as mechano-ceramic nature, rich chemistry, and hydrophilicity, to name a few. However, one of the most challenging issues in any composite/hybrid system is the interfacial wetting. Having a superior integrity of a given composite system is a direct consequence of the proper wettability. While wetting is a fundamental feature, dictating many physical and chemical attributes, most of the common nanomaterials possesses poor affinity due to hydrophobic nature, making them hard to be easily dispersed in a given composite. Thanks to low contact angle, MXenes can offer themselves as an ideal candidate for manufacturing different nano-hybrid structures. Herein this review, it is aimed to particularly study the wettability of MXenes. In terms of the layout of the present study, MXenes are first briefly introduced, and then, the wettability phenomenon is discussed in detail. Upon reviewing the sporadic research efforts conducted to date, a particular attention is paid on the current challenges and research pitfalls to light up the future perspectives. It is strongly believed that taking the advantage of MXene’s rich hydrophilic surface may have a revolutionizing role in the fabrication of advanced materials with exceptional features.

## Highlights

- The wetting behavior of 2D materials, MXenes in particular, is presented.
- Owing to rich chemistry, MXenes have great potentials to be employed in various composite/hybrid systems.
- Hydrophilicity and superior physical properties make the MXenes a great reinforcing agent.S

## Graphical abstract

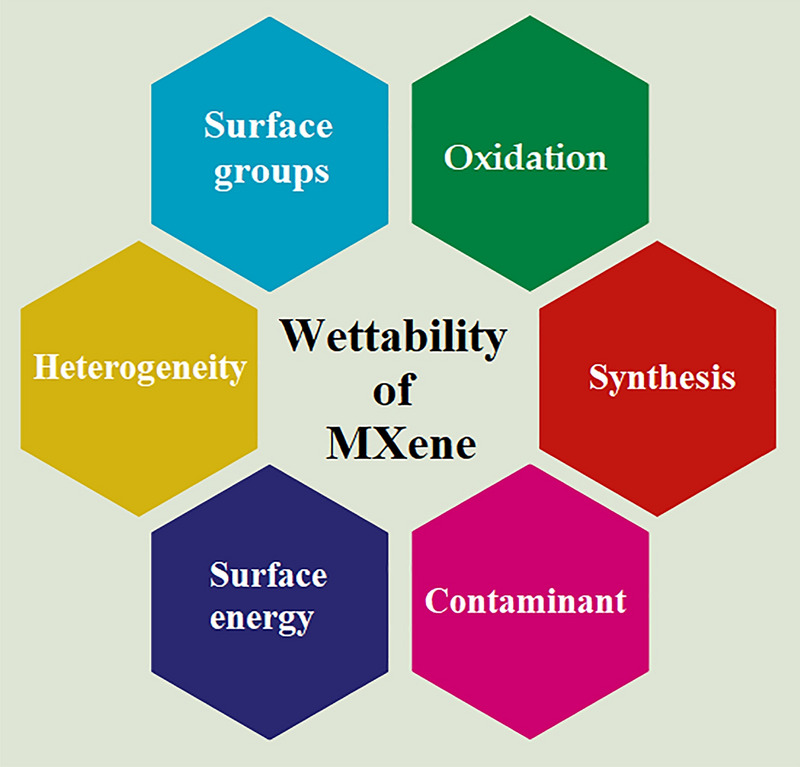

## Introduction

Since the discovery of MXene in 2011, MXenes have extensively been employed in a variety of fields ranging from composite materials [1], energy storage and catalytic conversion [2, 3], electromagnetic and electronics [4], environmental and sensing [5], biomedical [6, 7], and mechanical applications [1] to those basic and fundamental studies [8]. Furthermore, in many applications, MXenes are usually used with other materials to produce hybrids or composites, particularly when one of the final goals is to mechanically reinforce or electrically conduct the composite materials. In these applications, a reinforcing agent should be properly wetted by a given host material leading to a desired physical integrity; however, most of the common nanomaterials usually have a hydrophobic surface making them hard to be easily dispersed in a variety of matrices of polymers, metals, ceramics, etc. [9]. Further, owing to great contact angles of these poorly dispersed nanomaterials, the bonding strength in the interfacial zone tends to diminish when the given nanomaterial is embedded in a hybrid/composite system. For instance, nano-carbides, -ceramics, -nitrides, and -oxides are poorly wetted by liquid metals like aluminum alloys owing to hydrophobic nature of the filler materials. Similar phenomenon has also been reported in polymer composites as the particles tend to agglomerate due to interparticle forces such as van der Waals. According to the literature, such poor wetting and inadequate surface hydrophilicity has been a great bottleneck in nanocomposites; extensive research efforts have been made to date to address the mentioned challenge; strategies such as the coating of nanomaterials, thermal methods [10], or additionally mechanical treatments have been developed to fight against the inherent hydrophobic nature of the common nanomaterials, all trying to boost the nanomaterial matrix affinity [9, 11–13].

The aforementioned techniques aim to change the surface interfacial interactions, such as increasing hydrophilicity and surface energy in the mating regions. However, instead of applying time-consuming, costly, and exhausting processing’s, it may be better to identify a nanomaterial with inherent hydrophilic behavior so can be easily de-agglomerated, dispersed and homogenously distributed in a given host matrix and could establish a strong bonding in the corresponding interface.

MXene is a newly developed 2D hydrophilic nanomaterial with a general formula of M_n+1_X_n_T_x_, comprising n + 1 layers of group elements of 3–6 early transition metals, interleaved by n layer of carbon and/or nitrogen atoms; the surface terminations such as –F, –OH, and –O are represented by T_x_ [14]. Ti_3_C_2_T_x_ is the first member of MXene family being studied in different fields and varied applications. Having a combination of excellent physical and chemical properties such as 24,000 S cm^−1^ electrical conductivity in Ti_3_C_2_T_x_ MXene [15], 386 GPa Young’s modulus in Nb_4_C_3_T_x_ MXene [16], and a relatively lower water contact angle (e.g., 21.5° in MXene films [17]) enables this hydrophilic 2D flake to be used in critical applications ranging from supercapacitors to composite manufacturing. MXenes are synthesized through the selective etching of MAX precursor materials of M_n+1_AX_n_ general formula with A denoting to the elements of 11–16 groups like Al [18]. The resultant weakly bonded etched structure is then exfoliated mechanically to produce MXene. Upon accomplishment of the etching process, the metallic surface of the MXenes can quickly be saturated by a variety of terminating groups ranging from –F, –OH, and –O to other halogens such as –Cl and –I, chalcogens such as –Te, –S, –Se, and even also –NH functionalization groups, bestowing a quite tunable and rich surface [19].

MXene possess a very high aspect ratio, with tunable T_x_ functional groups. The wetting characteristics of MXene-reinforced composite/hybrid materials still need further investigations. Herein, the fundamental aspects of wetting phenomenon are firstly introduced, and the mechanisms of wetting in composite materials are elaborated. The wettability of MXenes in a variety of materials like polymers is then deliberated with a particular attention on surface energy, contact angle, and the values of wetting parameters in different media. Finally, the research efforts in this regard are critically reviewed, and the current challenges and research voids are presented to clear up the future horizons.

## Wetting Basics

Wettability is a measurement of the interfacial interaction between a liquid and another liquid or solid. It is the tendency of a liquid material to keep contact with the surface of a solid material being governed by a force balance between adhesive and cohesive intermolecular interactions. Several important parameters such as the surface roughness and the composition of solid, drop size and history (i.e., evaporation) temperature, surface tension, the degree of contamination, as well as the gas pressure and composition affect the contact angle and hence the wetting conditions. It is believed that the wetting angle varies when the size of the nanomaterial changes; for instance, attaining the contact angle of 15°–60° for diethylene glycol and 24°–67° for deionized (DI) water when increasing the size of nano-indium oxide from 14 to 620 nm, being attributed to higher surface free energy of smaller sized particles as one of the main reasons while there might be also other mechanisms such as surface roughness and the distribution of nanoparticles [20].

**Fig. 1:**
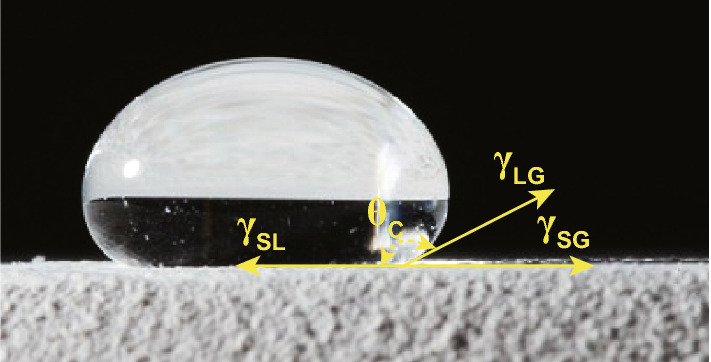
Young’s model of wetting with three vectors of solid–gas, solid–liquid, and liquid–vapor surface tensions, reprinted from Ref. [9]

A number of experiments such as static or dynamic sessile drop tests, pendant drop, or Wilhelmy method, to name a few, can be deployed to measure the wettability. Sessile drop method is more common due to a few important reasons such as simplicity and cost-effectiveness to name a few. According to Young’s model, schematically shown in Fig. 1, the contact angle is determined by Eq. (1) quantifying the wetting property of a solid by a liquid material [21]. In this equation, ${\gamma }_{\mathrm{SG}}$, ${\gamma }_{\mathrm{SL}}$, and ${\gamma }_{\mathrm{LG}}$, respectively, denote to solid–gas, solid–liquid, and liquid–vapor surface tensions while ${\theta }_{\mathrm{C}}$ being the contact angle. It is noted that Young’s model ignores surface texture effect as well as the gravity.

1 $$ \gamma_{{{\text{SG}}}} - \gamma_{{{\text{SL}}}} - \gamma_{{{\text{LG}}}} \cos \theta_{{\text{C}}} = 0 $$

Based on Eq. (1), raising the solid–liquid interfacial energy, increasing solid surface energy, and reducing liquid surface tension, all diminish the contact angle in solid–liquid interface being vital for better wettability.

To take into account the effect of volumetric dependence of the contact angle, the following modified Young’s equation was proposed by Gibbs wherein $\kappa$ represents the line tension, and $a$ is the droplet radius [9]:

2 $$ \cos \theta_{{\text{C}}} = \frac{{\gamma_{{{\text{SG}}}} - \gamma_{{{\text{SL}}}} }}{{\gamma_{{{\text{LG}}}} }} + \frac{\kappa }{{\gamma_{{{\text{LG}}}} }}\frac{1}{a} $$

Further, Eq. (3) considers the effect of Laplace pressure being ignored in the Young’s and the modified Young’s relation [9]:

3 $$ \cos \theta_{{\text{C}}} = \frac{{\gamma_{{{\text{SG}}}} - \gamma_{{{\text{SL}}}} }}{{\gamma_{{{\text{LG}}}} }} + \frac{\kappa }{{\gamma_{{{\text{LG}}}} }}\frac{1}{a} - \frac{\gamma }{{\gamma_{{{\text{LG}}}} }}\left[ {2 + \cos \left( {\theta_{{\text{C}}} } \right) - 2\cos^{2} \left( {\theta_{{\text{C}}} } \right) - \cos^{3} \left( {\theta_{{\text{C}}} } \right)} \right] $$

Contact angle is a unique property for a given solid, liquid, and vapor system; however, it varies in reality between the two advancing (maximal) and receding (minimal) values due to contact angle hysteresis [22]. Being measured by dynamic experiments wherein a droplet is in movement, the advancing ($\theta_{{\text{A}}}$) and receding ($\theta_{{\text{R}}}$) contact angles can be obtained, and their difference ($\theta_{{\text{A}}} - \theta_{{\text{R}}}$) provides the value of contact angle hysteresis, being greatly dependent on a number of parameters such as chemical heterogeneity and roughness [11, 22, 23].

Surface energy of a liquid–solid system is of great importance, dictating the wettability of a given material in a host material. According to LW–AB model, the total surface energy ($\gamma$) can be estimated through Eq. (4) that consists two important components, namely, dispersion component ($\gamma^{{{\text{LW}}}}$) and acid–base component ($\gamma^{{{\text{AB}}}}$):

4 $$ \gamma = \gamma^{{{\text{LW}}}} + \gamma^{{{\text{AB}}}} $$

The first component is due to Lifshitz–van der Waals interactions while the second is due to Lewis interactions [22].

## Wetting of 2D Materials Beyond MXenes

As mentioned earlier, most of the nanomaterials are inherently hydrophobic; the wetting of materials commonly diminishes when the particle size is decreased. It should be noted that the contact angle of a given material can be different under varied testing and environmental conditions [24].

As one of the most important 2D materials, graphene has shown superior mechanical properties; however, this nanosheet has a poor wetting behavior with different materials; a water contact angle of more than 95°–127° has usually been reported in the literature, exhibiting a hydrophobic surface characteristics of graphene [11, 25, 26]. Based on water contact angles measured by Wang et al. [23], it has been observed that the wetting angle of mono-layer graphene sheet is influenced by the substrate; they also measured the contact angle of 98.3° for graphite and 127° for graphene while a value of 90°–95° has been reported by Shin et al. [27] and Rafiee et al. [28] for the graphite. Later on in 2012, Shih et al. [29] developed a theory to model the van der Waals interactions between the liquid and graphene wherein a water contact angle of 96° on a mono-layer graphene was achieved. Similar water contact angle on mono-layer graphene was measured as 95°–100° by Taherian et al. [25], wherein it was believed that the main contribution to the work of adhesion is the water–substrate interaction energy. As seen, there is a relatively large amount of discrepancy in the reported contact angles that can be attributed to many parameters such as material variables, environment, and so forth. Apart from graphene, its derivatives such as graphene oxide (GO) have also been used in assorted applications; oxygen-containing groups in GO, GO-based bulk materials can be tuned to provide different wetting behaviors. From a typical treatment, the GO displays a hydrophilic property with the contact angles of about 30°–60°, modulating the structure and properties of GO and GO-based composites/hybrids [30]. According to the study of Wei et al. [30], those naturally wrinkles and oxidized patterns on GO considerably may affect the pinning effect and wetting state of the GO and its hybrids.

A different class of 2D materials, being extensively employed in a wide variety of research fields, is transition metal dichalcogenides (TMDs) [31]. As a layering van der Waals solid like MoS_2_, the transition metal occupies octahedral coordinates and trigonal prismatic coordinates, respectively, in IT and 2H structure, sharing edges with the closest neighbors within each flake to create a hexagonal honeycomb-like form. According to a number of research efforts made by, for example, Singh et al. [12] and Luan et al. [32], MoS_2_ films display a hydrophobic surface behavior despite its considerable surface charges and comparatively robust strength of van der Waals. An advancing contact angle of 98.0° and receding angle of 55.40° revealed considerable hysteresis in MoS_2_, probably due to the surface heterogeneity; further, the wetting angle tended to increase when the number of nanosheets increased [12]. Chow and colleagues [33] reached to a similar result since the water contact angle of mono-, bi-, and tri-layer MoS_2_ was 83°, ∼85°, and ∼90°, respectively, indicating the water interaction with the underlying substrate in wetting test of mono-layer TMD nanosheet. It is worth noting that the wetting behavior of MoS_2_ is greatly dependent on contamination, as it was seen the water contact angle of this nanosheet tends to change from ~ 69° (in isolated state) to ~ 89° (ambient condition) owing to organic contamination [34].

As seen, most of 2D nanomaterials usually exhibit a hydrophobic surface behavior, and their dispersion would be relatively challenging due to poor wettability, instigating many challenging issues in different composite/mixture/hybrid systems. For instance, in metal matrix nanocomposites, the poor dispersibility of common nanomaterials almost always results in micro- or nano-structural voids, imperfect interface, porosities, agglomeration, and inhomogeneous dispersion of the nano-filler in its host material [9, 35]. However, a newly developed 2D nanomaterial termed MXene has a hydrophilic nature being an evolutional discovery in nanocomposite field since it can be easily wetted by a variety of materials and hence establishing a strong interface/interphase in the mating region between the filler and the corresponding matrix. The next section discusses the wetting behavior of MXene with the research efforts reported to date in the literature.

## Wetting of MXenes

**Fig. 2:**
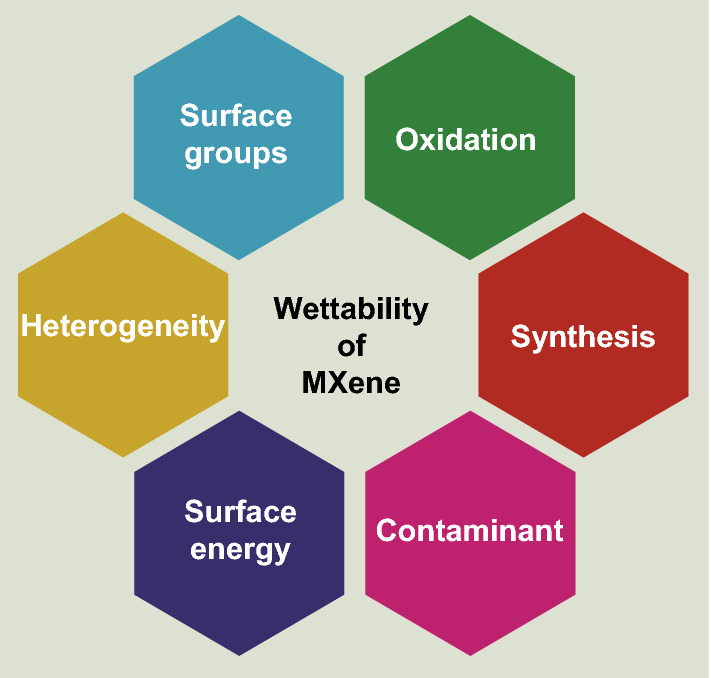
Wettability of MXene with key influencing parameters

Wettability is of crucial importance for almost all composite systems as it is governing the interfacial characteristics to a large extent. Unlike the above-discussed 2D materials, MXene has a relatively lower contact angle, hydrophilic surface, and hence great dispersibility in a variety of solvents and composites. In this section, the wetting of MXene is briefly reviewed and critically discussed based on the key parameters with some of them mentioned in Fig. 2. Oxidation, surface groups, heterogeneity, synthesis methods, contaminants, and surface energies are just a number of important aspects with the wetting behavior of MXenes in composite settings [36].

First of all, it is strongly believed that the preparation process and condition of MXenes may greatly affect the wettability of MXenes, owing to different parameters such as the termination groups being appeared on the MXene surface depending on the synthesis method [22, 37]. The presence of impurities and those remaining MAX phase particles in the final MXene powder may affect the wetting properties as well.

Surface energy is one of the most governing indications of wetting. While the surface energy of graphene and graphene oxide is ~ 46.7 and ~ 62.1 mJ m^−2^, respectively, the value of surface energy of Ti_3_C_2_T_x_ MXene was measured as about 64.48 mJ m^−2^, being capable of forming strong adhesive bonding’s with materials like polymers [23, 27, 38]. The value of 64.48 mJ m^−2^ for the surface energy of the MXene flakes is the implication of good adhesion between the flakes and the epoxy matrices, commonly having the surface energy of ~ 44.4–59.5 mJ m^−2^. According to Zukiene and colleagues [38], the surface energies of MXene sheets considerably affect the hydrophilicity and wetting behavior of MXene-based hybrids. The values of surface energy of 10-layer MXene coatings were measured and estimated to be in the range of 47.98 and 64.48 mJ m^−2^. It was also calculated that the Ti_3_C_2_T_x_ has the work of adhesion of up to 123.6 mJ m^−2^ with the epoxy material, being promising for composite manufacturing.

MXene’s wetting angle varies when wetted by different media, which means that the contact angle may be influenced by substrate. For instance, the contact angles of 48.50°, 29.95°, and 47.27° were measured for 10-layer MXene when the wetting liquids have, respectively, been chosen as water, diiodomethane, and glycerol [38]; the chemistry of both wetting liquids, MXene film, as well as the interactions between the two aforesaid items, i.e., liquid and MXene, is to be well considered.

Similar to the results obtained from the other 2D materials such as graphene or MoS_2_, it was also seen that the wetting behavior is dependent on the thickness of MXene films or the number of stacked layers [11, 32]. For instance, according to Raj et al. [11], the receding contact angle on two investigated substrates was proved to be dependent on the number of graphene layers being attributed to the intrinsic defects introduced during the growth and transfer processes. Investigations revealed that the contact angle varies with the number of MXene layers. It has been experimentally examined that the water contact angles were about 45°, 66°, and 68°, respectively, for 1-, 5-, and 10-layer MXenes [38]. The water contact angle sharply increased from 1- to 5-layer sample but then does not change considerably as the values associated with 5- and 10-layer samples are not significantly different, probably owing to the interaction of liquid molecules with that of underlying material.

The effect of roughness on the wetting behavior of MXene seems to be inevitable as it has frequently been reported in the literature; rougher surfaces may be better wetted [38]. The water contact angles of Ti_3_C_2_T_x_ MXene films coated on glass substrates and the average roughness of 11.98, 37.88, and 84.93 nm were measured as 68.40°, 66.20°, and 48.50°, respectively; similar trends have been observed when other wetting liquids such as diiodomethane and glycerol were used owing to the higher surface roughness and surface area of the exposed hydrophilic MXene nanosheets [18, 38]. Polar groups on the MXene surface affect the wetting angle; as it was, for example, measured as 36° for the Ti_3_C_2_T_x_ while it decreased to 30° when the same experiment was performed on the oxidized Ti_3_C_2_T_x_ [39]. It can be concluded that the surface polar groups may change the interactions between the MXene sheets with that of molecules of wetting liquids.

**Fig. 3:**
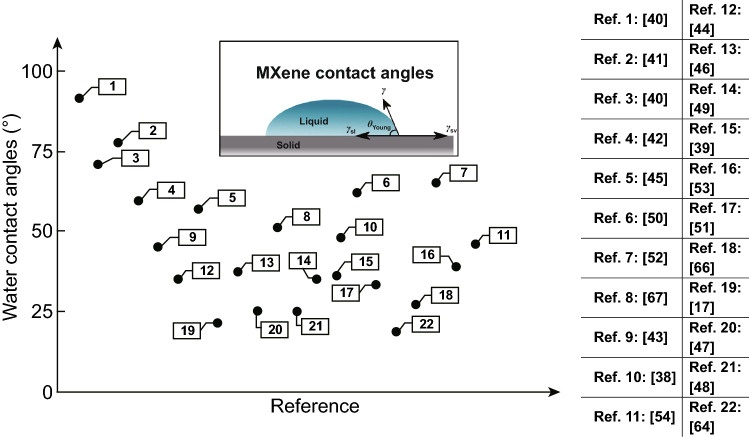
Wetting angle values reported in the literature

Apart from the above-discussed parameters influencing the wetting features, it appears a relatively high scattered contact angle values which have already been reported for MXenes [17, 38–63]. The first attempt to measure the contact angle value of MXene was performed by Ghidiu et al. [17] in 2014 wherein a 21.5° wetting angle was measured by a water droplet on the rolled Ti_3_C_2_T_x_ MXene films of < 100-µm thickness, confirming the hydrophobicity of 2D MXenes. Since then, several research efforts have been made to well-understand the wetting behavior of MXene, underlying mechanisms and the key parameters influencing the wettability of MXene in different media. Contaminants, intercalated ions, as well as underlying substrates are thought to be some of the reasons of discrepancy in the contact angle values observed and reported in the literature for the MXene sheets or even for the other well-studied 2D materials like graphene [58], indicating that there are exist a great gap of knowledge for understanding the wetting behavior of the mentioned nanomaterials and their films, MXene in particular. Figure 3 depicts the contact angle of MXene sheets reported in different research studies; as seen, the scattering in the contact angles is relatively large, ranging from 18.6° to 91° [64, 65]. Considering the degree of discrepancy demonstrated in Fig. 3, it is believed that there may exist a variety scattering sources.

According to Zhou et al. [58], contact angle may change in time due to the stored conditions; for example, the water contact angle of those Ti_3_C_2_T_x_ MXenes dried in a 60° oven had the highest scattering of about 27° while it was only 22° for the nanosheets dried naturally; finally, nearly no scattering was detected for the contact angle of the samples dried in vacuum condition, demonstrating the inevitable oxidation effects (being often heterogeneously occurred) on the contact angles and scattering behavior when stored in air, as confirmed by XRD analysis. Moreover, as an influencing source of disrupting inherent wetting behavior, it was found that organic contaminants, commonly absorbed from air, may cause scattering as well [58].

Studying the existing reports on the wetting angles, it has been seen that a list of variables associated with synthesis and preparation process, film or coating techniques and its corresponding parameters, storage and maintenance conditions, substrate, contaminants, oxidations, wetting liquid, surface chemistry and topography, the accuracy of measuring techniques, the number of layers, as well as the thickness, among others, are all affecting this parameter to some extent. If the mentioned variables are correctly chosen, the superior wetting is expected. For example, getting 400% improvement in the yield strength of only 0.3 wt% Ti_3_Si_0.75_Al_0.25_C_2_ filled PMMA unmask the outstanding wetting and interfacial affinity in the mentioned composite system [55]; without having enough hydrophilicity, it is believed that such an improvement in mechanical response is quite unlikely. Having great surface energy and the presence of functional groups such as hydroxyls or carbonyls can provide strong adhesion at the interfacial region. In the studies by Monastyreckis et al. [57] or Kilikevičius et al. [59], the reinforcing MXene particles could dramatically enhance the interface and overall mechanical performance, owing to a few important underlying mechanisms, namely: (1) Mechanical interlocking boosted by hydrophilic nature, this kind of nanomaterials can act as an obstacle against the crack growth or propagations and (2) chemical bonding’s between MXene surface and its terminating groups with that of host matrix surface may provide strong bonding’s between the filler and host materials. In a computational effort, it was revealed that the Young’s modulus of 30 vol% MXene-based composite could be greatly enhanced by ~ 740%, compared to its neat samples [57].

**Fig. 4:**
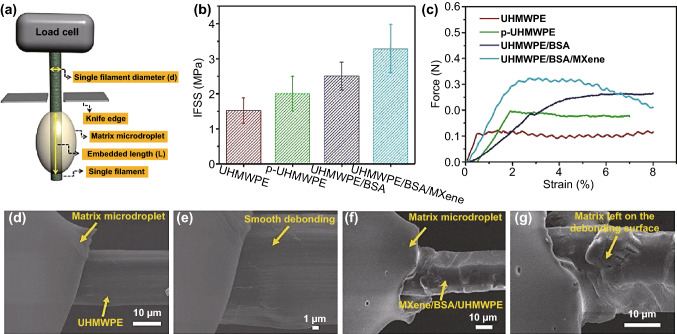
**a** Pull-out test schematic of single carbon fiber; **b** the values of interfacial shear strength (IFSS); and **c** variation of force versus displacement in different conditions; the debonded part of the neat UHMWPE fiber at **d** low and **e** high magnifications. The debonded part of UHMWPE/BSA/MXene fiber at **f** low and **g** high magnifications. Reprinted with permission from Ref. [61]

Apart from the use of MXenes as the main reinforcing agent, they can also act as a wetting promoter, improving the wetting behavior of other reinforcing materials through coating to enhance their interfacial strength. For example, Ying and co-workers [60] could improve the shear strength of carbon fiber (CF)-reinforced epoxy matrix by 186% when the CFs were coated by Ti_3_C_2_T_x_ MXene nanoflakes (see Fig. 4); physical interlocking through the modified surface topography as well as the strong hydrogen bonding interactions between the carboxyl groups of carbon fibers and the functional groups of the MXene were thought to be the main playing role strengthening mechanisms. Also, due to hydrophilicity and wettability of MXenes, they can promote the penetration of epoxy matrix into the surface microstructure of the reinforcing agent [61]. As shown in Fig. 4, the pull-out experiment of UHMWPE/BSA/MXene nanocomposite exhibited a strong interphase as the failure seems to be occurred at the matrix and not necessarily at the interphase between the reinforcing agent and matrix; debonded fibers were seen to be mostly covered by the epoxy material, indicating a mechanically and chemically strong bonding originated by the well-wetted MXene sheets, attaining a 116% improvement in the interfacial shear strength of the final composites.

Recently, Machata et al. [22] investigated the advancing and receding contact angles of Ti_3_C_2_T_x_ MXene films; a giant contact angle hysteresis of ~ 44°–52° measured from MXene films was thought to be due the chemical heterogeneities, arising from a variety of functional groups of –O, –F, and –OH; the effect of underlying substrate was found negligible the water droplet, and the underlying substrate caused by imperfect MXene coatings.

Based on the study of Zhang et al. [56], it was found that Ti_3_C_2_ MXene sheets are well-wetted by UHMWPE polymer chains, and hence, a strong interfacial bonding is achieved; the incorporation of MXene nanoflakes could enhance crystallization and improve mechanical and tribological properties. Owing to a superior affinity between the MXene particles and the polymer matrix, the load transfer from the matrix to filler materials has successfully been achieved.

**Fig. 5:**
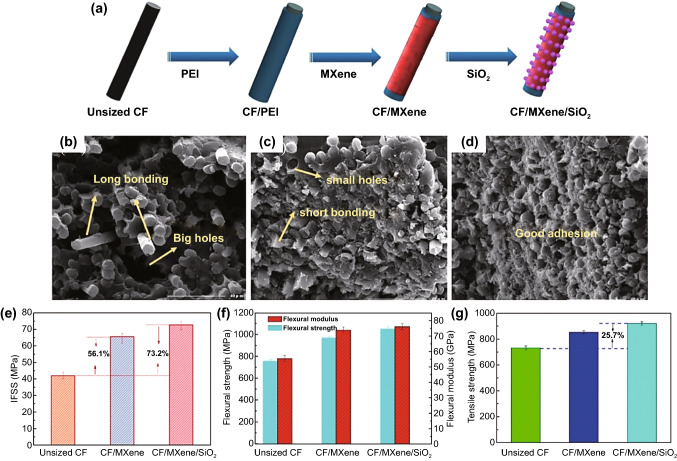
**a** Schematic MXene/SiO2 3D structure onto carbon fiber, the fracture surface of the **b** unsized CF/EP, **c** CF/MXene/EP, and **d** CF/MXene/SiO2/EP nanocomposites. The values of **e** interfacial shear strength (IFSS), **f** flexural strength, and **g** flexural modulus of MXene/SiO2-reinforced composite materials. Adopted with permission from Ref. [62]

Apart from the possible application of MXene as the reinforcing material or wetting promoter, MXenes can be employed in those composite materials wherein the embedded reinforcement is poorly wetted by its host matrix. For example, silica is poorly wetted by either polymer or metal matrices [66–68]. However, the combination of silica nanoparticles and Ti_3_C_2_T_x_ MXene nanosheets was seen to enhance the interfacial strength of the carbon fiber (CF)-reinforced epoxy resin composites to a great extent [62]. According to Guo et al. [62], the surface energy of inert surface glazy CF could be considerably boosted from 26.67 to 48.12 mJ m^−2^ which, in turn, resulted in an enhanced interfacial shear strength by ~ 73.2% compared to the neat matrix material as demonstrated in Fig. 5; dynamic mechanical analysis (DMA) revealed a 64% increase in storage modulus of the composite material, reaching to 33.55 GPa. Decoration of carbon fibers with silica and MXene particles might create a 3D cross-linked structure, limiting the mobility of epoxy resin and changing the failure type from adhesive to cohesive behavior.

**Fig. 6:**
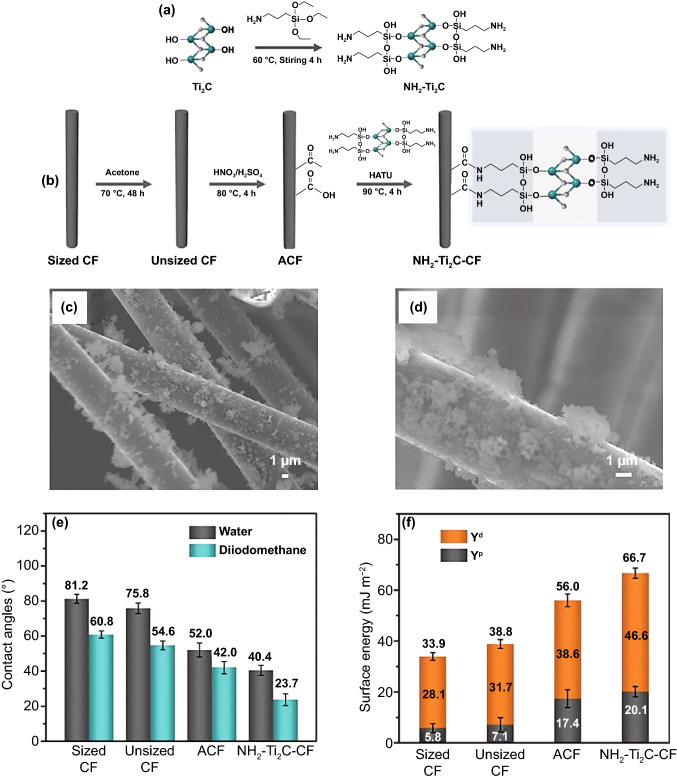
**a** Schematic of Ti2C modification and **b** NH2–Ti2C grafted on the surface of carbon fibers. SEM morphologies of CFs decorated by **c** NH2–Ti2C–CF, with **d** higher magnification. **e** Contact angle values and **f** surface energy values of different CFs. Adopted with permission from Ref. [63]

Similar research effort on the surface modification of carbon fiber was followed by Ding et al. [63] wherein Ti_2_C MXene sheet was modified by 3-aminopropyl triethoxysilane and then grafted on carbon fiber-reinforced epoxy matrix, leading to a 78% improvement in interfacial shear strength owing to mechanical interlocking and uniformly grafted MXene nanoflakes onto the fibers through strong covalent bonding. Schematic diagram of Ti_2_C modification and the grafted carbon fibers are shown in Fig. 6. It is evident that the Ti_2_C coating layer could considerably enhance the surface energy; consequently, the contact angle drops from 81.2° for the neat matrix to the value of 23.7° for the composite sample, attaining ~ 70% reduction in contact angle providing much better wettability, as confirmed in surface energy as well, wherein the dispersion component increases from 33.9 mJ m^−2^ for the neat material to 66.7 mJ m^−2^, reaching to a ~ 96% higher surface energy.

## Summary and Perspective

One of the most important issues in any composite manufacturing systems is wetting. The affinity between a given filler with its corresponding host matrix material directly affects the interphase or interfacial region and hence a lot of physical properties such as mechanical or electrical performance. Most of reinforcing nanomaterials are poorly wetted in a given matrix due to their hydrophobic nature; being frequently confirmed for common 2D materials such as graphene or graphene oxides. However, investigations revealed that MXenes, a class of novel 2D nanomaterials, is mostly hydrophilic and can be easily dispersed in a variety of solvents and/or other materials like polymers. Having excellent surface properties due to hydrophilicity with tunable surface functionalization in one hand and a superior mechano-ceramic nature of MXene in other hand makes this material is a great asset as the other common nanomaterials are poorly wetted by a variety of materials.

To date, little is known about the wetting properties of MXenes, and hence, extensive research efforts are required to fully understand the different aspects of this topic. The scattering of the wetting angles reported in the literature is large as quite sporadic research efforts have been conducted so far. It is believed that wetting of 2D nanomaterials like MXene is sensitive to different parameters such as surface heterogeneity that should be investigated further. The effects of contaminants and synthesis process on the wetting of MXenes should be further studied. The uniformity of MXene layers should be considered, and the possible effects on the contact angle shall be estimated. At the meantime, there exist a lot of unanswered questions that the effect of particle size, type of the termination groups, and the degree of oxidation being only a few instances. The effect of contaminants or even remaining MAX phase particles in the MXene is of crucial importance, and their positive/negative consequences on the wettability should be categorized and rationalized. The effect of remaining water molecules between the MXene layers has not yet been fully investigated, and no comprehensive research is available regarding the effect of time of contact on the final wetting angles. Apart from the effect of MXene synthesis processes which has a dominant influence on the final wetting performance, the effect of coating technique and preparing MXene films on a given substrate seems to influencing. The chemical interactions between a given substrate-MXene system may greatly affect the affinity and hence final physical properties. Finally, there exist several controversial discussions about the influencing parameters; for example, a number of research efforts reported roughness [18, 38] as the main reason of contact angle hysteresis, while in other publication, chemical heterogeneities might be the reason (or the most important reason) of hysteresis. Therefore, further investigations are demanded to develop standard experiments to measure the correct contact angle and to rationalize the differences reported in the literature.

It is worth noting that although the wetting of MXene in polymeric materials has not been fully understood, the wetting/affinity of MXenes in ceramics or metals is quite unknown, and no research efforts, to best our knowledge, have yet been reported, focusing on the wetting of such systems. Common reinforcing agents such as graphene are poorly wetted by metals, and then, the key issues poor dispersibility, non-homogenous distribution, agglomerations, imperfect bonding at the interface, and porosities, among others, have always been a bottleneck in this field. Like the promising applications of MXenes in polymeric-based hybrid/composite materials, it is believed that the addition of MXenes to metals or ceramics may result in surprising results as the MXenes are both hydrophilic and mechanically robust, capable of enhancing microstructural features of advanced novel composite materials and structures.
